# Impact of Alignments on the Accuracy of Protein Subcellular Localization Predictions

**DOI:** 10.1002/prot.26767

**Published:** 2024-11-22

**Authors:** Maryam Gillani, Gianluca Pollastri

**Affiliations:** ^1^ School of Computer Science University College Dublin (UCD) Dublin Ireland

**Keywords:** bioinformatics, BLAST, convolutional neural networks, deep learning, HHblits, protein sequence alignments, protein subcellular localization predictions, subcellular localization predictions

## Abstract

Alignments in bioinformatics refer to the arrangement of sequences to identify regions of similarity that can indicate functional, structural, or evolutionary relationships. They are crucial for bioinformaticians as they enable accurate predictions and analyses in various applications, including protein subcellular localization. The predictive model used in this article is based on a deep – convolutional architecture. We tested configurations of Deep N‐to‐1 convolutional neural networks of various depths and widths during experimentation for the evaluation of better‐performing values across a diverse set of eight classes. For without alignment assessment, sequences are encoded using one‐hot encoding, converting each character into a numerical representation, which is straightforward for non‐numerical data and useful for machine learning models. For with alignments assessment, multiple sequence alignments (MSAs) are created using PSI‐BLAST, capturing evolutionary information by calculating frequencies of residues and gaps. The average difference in peak performance between models with alignments and without alignments is approximately 15.82%. The average difference in the highest accuracy achieved with alignments compared with without alignments is approximately 15.16%. Thus, extensive experimentation indicates that higher alignment accuracy implies a more reliable model and improved prediction accuracy, which can be trusted to deliver consistent performance across different layers and classes of subcellular localization predictions. This research provides valuable insights into prediction accuracies with and without alignments, offering bioinformaticians an effective tool for better understanding while potentially reducing the need for extensive experimental validations. The source code and datasets are available at http://distilldeep.ucd.ie/SCL8/.

## 1 Introduction

Alignments refer to the arrangement of sequences (such as nucleotide or amino acid sequences) in a way that highlights their similarities, differences, and structural characteristics [1]. In bioinformatics, alignments are commonly used to compare biological sequences with each other or with a reference sequence. The primary goal of sequence alignment is to identify similarity regions, which may indicate functional, evolutionary, or structural relationships between sequences [2]. There are two main types of sequence alignments: pairwise alignment and multiple sequence alignment (MSA). Pairwise alignment involves aligning two sequences with each other to identify regions of similarity or homology [3]. This type of alignment is often used to compare sequences from different individuals, species, or homologous genes. However, MSA involves aligning three or more sequences simultaneously. MSA is particularly useful for comparing homologous sequences from multiple related species or genes within the same organism. It helps identify conserved regions, motifs, and structural features shared among sequences [4].

Alignments play a crucial role in protein subcellular localization predictions. There are mainly two types of subcellular localization prediction methods i.e. homology based methods (template‐based) and Ab initio based methods (template free) [5]. In template‐based methods, alignments are used to identify the most suitable template protein structures or sequences for modeling the query protein. It helps in subcellular localization predictions by providing structural and functional insights into localization signals or motifs present in template proteins [6]. However, template‐free methods do not rely on homologous templates for initial modeling. Therefore, alignments can aid in the identification of sequence motifs or patterns associated with specific subcellular localizations [7]. By comparing the query protein sequence to sequences of proteins with known localizations, alignments can help identify conserved motifs or sequence features that may serve as localization signals. These motifs can then be used as features to predict the subcellular localization of the query protein [8].

Although alignments may not be as central to ab initio methods as they are to homology‐based methods, they can still provide valuable information about sequence‐structure relationships, conservation patterns, and functional motifs relevant to subcellular localization prediction [9]. By leveraging alignment‐based information, ab initio methods can enhance the accuracy and reliability of subcellular localization predictions for novel protein sequences. Precisely, alignments can still play a role in a more limited capacity compared with methods that use homology‐based information [10]. While ab initio methods primarily rely on sequence information, alignments with structurally and functionally characterized proteins can provide insights into the potential structural and functional properties of the query protein. Certain subcellular localizations may be associated with specific structural/functional features or domain arrangements, which can be inferred from alignments with proteins of known structure [11].

The challenges of not including alignments in protein subcellular localization predictions include limited sequence information, difficulty in identifying localization signals, reduced discriminative power of predictive models, limited contextual information, and decreased prediction accuracy overall [12]. Therefore, alignments play a critical role in enhancing the accuracy and reliability of subcellular localization predictions by providing valuable information about sequence‐structure–function relationships and conservation patterns [13]. Followed by the aforementioned challenges, the highlighted contributions, objectives, and motivations of this article are as follows:

This article conducts a comparative analysis of subcellular localization prediction methods with and without alignments. While previous studies may have explored the role of alignments in different aspects of bioinformatics, such as protein structure prediction and subcellular localization predictions, this research fills a gap by specifically focusing on alignments impact on localization prediction accuracy. This article is comparing with and without alignments predictions accuracy through extensive experimentation, which can facilitate early career bioinformaticians to gain a deeper understanding of how alignments contribute to prediction accuracy and how different factors influence the performance of prediction models. Also, experimentation with alignment‐based and alignment‐free methods encourages early career bioinformaticians to think critically and creatively about the challenges and limitations of each approach.

While alignments are widely used in various bioinformatics applications, their specific role in subcellular localization prediction may not have been thoroughly explored. This study fills a gap in knowledge by systematically evaluating the importance of alignments in improving prediction accuracy, providing valuable insights for researchers and practitioners in the field. Moreover, to the best of our knowledge, there has been no prior investigation into the specific problem addressed in this study. Despite the extensive literature on subcellular localization prediction and the importance of sequence alignments in bioinformatics, the direct comparison of prediction accuracy with and without alignments in this context appears to be novel. Our study fills this gap by providing a comprehensive analysis of the impact of alignments on subcellular localization prediction accuracy.

The rest of the paper is organized as follows: Section 2 covers the Literature review with a Table that covers the latest alignment tools. Section 3 discusses detailed methodology with the predictive model used. It also covers with alignments and without alignments protocols used in experimentation. Section 4 provides results and analysis with supporting graphs and tables. Section 5 concludes the paper.

## 2 Literature Review

The use of alignments in bioinformatics and computational biology, in protein subcellular localization prediction, has been a fundamental aspect of research for several decades [14]. Integration of alignments for prediction accuracy has been a longstanding practice in the development of protein subcellular localization prediction methods. There is an extensively vast literature present that uses protein subcellular localization prediction with alignments that are added through alignment tools [15]. For example, ML‐FGAT [16] uses PSI‐BLAST tool. MFannot [17] heavily use BLAST for sequence similarity with known genes. OrganelX [18] uses FASTA along with support vector machine (SVM) and multi‐class classifiers. VIRify [19] uses Blastn as well as mega‐blast. Blastn for nucleotide sequence analysis and mega‐blast for rapidly identifying highly similar sequences in large nucleotide databases. MULocDeep [20] uses BLAST, Blastp and PSI‐blast for various needs.

It is possible to achieve competitive prediction accuracy in subcellular localization prediction without using explicit sequence alignments [21]. Alignment‐free methods leverage alternative approaches, such as deep learning architectures, sequence‐based features, and machine learning algorithms, to effectively capture the information necessary for accurate prediction of protein localization patterns. Some tools may employ other types of data or features, such as protein sequence motifs, physio‐chemical properties, evolutionary information, or machine‐learning models trained on annotated datasets, to predict protein subcellular localization. For example, E‐MuLA [22], does not specifically use alignment tools. Rather, uses K‐nearest neighbors (KNNs), decision trees (DTs), and long short‐term memory (LSTM) classifier. Similarly, Graph‐BERT [23] is a hybrid protein subcellular localization prediction tool that uses a language model‐based framework for protein–protein interaction identification and does not necessarily add alignments. DeepLoc‐1.0 [24] uses the deep learning architecture (recurrent neural networks, RNNs) incorporates additional sequence‐based features for subcellular localization prediction and does not explicitly use alignments. RNNs are well‐suited for sequential data, such as protein sequences, as they can capture long‐range dependencies and context information.

DEEPred [25] is a deep learning‐based predictor that integrates various sequence‐derived features, including physio‐chemical properties, amino acid composition, and sequence motifs, to predict protein subcellular localization. While it does not explicitly use alignments. AdaPPI [26] also independently works without incorporating alignments through any particular tool. It is based on adaptive graph convolution networks in a protein–protein interaction network. SDN2GO [27] employs a hybrid model integrating convolutional neural network (CNN) with Bidirectional Gated Recurrent Unit (BiGRU), attention mechanism, augmented with protein language model embedding. It does not implicitly use any alignment tool. MSTLoc [28], utilizes multi‐scale deep learning to address the challenge of imbalanced multi‐label protein subcellular localization prediction using immunohistochemistry images. Also, TooT‐SC [29] apply BERT (bidirectional encoder representations from transformers) language model for protein sequences. TripletProt [30] is based on protein representation learning via Siamese neural networks. Rather than using any alignment tool, it focuses on capturing essential features in the representation learning of biological entities.

Indirect use of alignments may occur when alignment‐derived features are incorporated into machine learning models or when sequence similarity measures are used to infer functional annotations based on the similarity to annotated proteins with known subcellular localization. In such cases, alignments may not be the primary focus of the prediction method but may still contribute to the overall predictive accuracy. In short, some tools prioritize alignment‐based approaches, while others rely on alternative strategies to achieve accurate predictions. For example, SCLpred family of predictors SCLpred‐MEM [31], SCLpred‐EMS [31], and SCLpredT [32] offer tailored solutions for accurate subcellular localization prediction while using alignments (mostly from PSI‐Blast) and also relies on Deep N‐to‐1 CNNs. The alignment tools along with customized models make a perfect duo for better prediction accuracy. Another example is In‐Pero [33] uses PSI‐BLAST along with deep learning embedding of protein sequences to predict the localisation of peroxisomal proteins.

Adding alignments typically involves comparing a query sequence (e.g., protein sequence of interest) to one or more reference sequences (e.g., sequences in a database) to identify similarities, differences, and patterns of conservation [34]. This process can be performed using alignment algorithms and tools. There are numerous alignment tools available in bioinformatics, each with its own strengths, features, and applications. Categories of alignment tools along with relevant descriptions and suitable predictors examples with respect to novelty factors and distinguished features are given in the next section.

Alignment tools are classified to cover a broader set of five categories i.e. pairwise alignment tools, MSA tools, structure‐alignment tools, profile‐based alignment tools, and genome alignment tools explained as follows.

### 2.1 Pairwise Alignment Tools

BLAST (basic local alignment search tool) identifies regions of similarity between biological sequences (such as protein sequences) and provides statistical significance scores for these matches. Examples include SherLoc2 [35], mGOASVM [36], MULocDeep [20]. BLAST novel features include a combination of heuristic algorithms, flexible scoring systems, and statistical significance estimation.

FASTA, one of the earliest tools developed for sequence alignment and searching, utilizes a text‐based format commonly used to represent either nucleotide or protein sequences. Alongside FASTA, tools like PSORTm [37], SubLocEP [38], and PSORTb [39] are integral to the process of sequence similarity searching. These tools employ a word‐based indexing approach that focuses on local alignment, which is supported by a flexible scoring system to enhance the accuracy and efficiency of the search results.

SMI BLAST integrates elements of both local and global alignment algorithms, combining the precision of Smith–Waterman with the efficiency of BLAST. This hybrid approach is exemplified in tools like SMI‐BLAST [40], Cppsite 2.0 [41], and ProtRe‐CN [42], which utilize a selective alignment strategy to reduce computational time significantly. This methodology enhances the tool's utility in complex sequence analysis, providing both depth and breadth in alignment capabilities.

### 2.2 MSA Tools

Clustal Omega is an hidden Markov models (HMM)‐based tool that employs a progressive alignment strategy, which initially aligns sequences in pairs and then progressively aligns them with each other based on their similarity scores. This method is particularly effective for genome‐wide sequence analysis due to its scalability and efficiency. Notable implementations of this approach can be seen in applications such as BAliBASE [12], TargetP [43], and WoLF PSORT [44], each of which leverages the strengths of Clustal Omega to deliver enhanced sequence analysis capabilities.

ProbCons represents a consistency‐based approach to sequence alignment, leveraging evolutionary information, and probabilistic models to enhance alignment accuracy. This approach is exemplified in its application across various studies, including its own foundational research [45], as well as in benchmark datasets such as BAliBASE [12]. Further developments and comparisons with similar methodologies, like Probalign [46], highlight the efficacy of consistency‐based approaches and the adaptive use of probabilistic models to refine the precision of sequence alignments.

Kalign employs the Wu‐Manber string‐matching algorithm for aligning sequences and utilizes a progressive alignment approach to efficiently handle multiple sequences. This tool is particularly adept at leveraging parallel processing, which significantly enhances its performance and scalability. Noteworthy implementations of Kalign's methodology include its use in SIMD environments as detailed in [47], and its evolution through various versions such as the original Kalign [48] and Kalign‐LCS [49]. These adaptations highlight the tool's robustness and its capacity for handling large‐scale sequence alignments.

### 2.3 Structure‐Alignment Tools

DALI is a tool that compares protein structures by analyzing the distances between alpha carbons within those structures. This method facilitates global structure alignment and is implemented effectively in various platforms, including the Dali server [50], which provides comprehensive visualization tools and graphical interfaces for enhanced interpretability. Additional implementations, such as pHLA3D [51] and DaliLite [52], leverage DALI's robust framework to offer precise and visually accessible structural comparisons, making it invaluable for detailed protein analysis.

TM‐align specializes in comparing protein structures through the alignment of their alpha carbon backbones, enabling precise global structure alignment. This method is employed in various specialized adaptations, including Fr‐TM‐align [53], which incorporates template modeling score calculations, and J‐TMAlign [54], known for its integration with advanced visualization tools. Additionally, mTM‐align [55] extends the capabilities of TM‐align by enhancing its computational efficiency and accuracy, demonstrating the adaptability and utility of this alignment framework in detailed structural analysis.

MAMMOTH is a robust tool that employs a blend of sequence and structural information to detect conserved regions and structural motifs in proteins. This approach is illustrated in its application in various studies such as the original MAMMOTH tool [56], and further utilized in specific protein analyses like BcsG [57] and Sonic Hedgehog [58]. The method is characterized by its model‐based matching technique, which ensures the statistical significance of alignments and facilitates the creation of detailed 3D representations of aligned protein structures, enhancing both the depth and clarity of structural insights.

VAST (vector alignment search tool) assists in identifying and visualizing similarities between protein structures. It compares protein 3D structures using vector alignments of their backbone elements, highlighting both exact matches and similar conformations. VAST [59], MMDB, and VAST+ [60], VAST [61] integrating VAST enhances the robustness of predictions by leveraging structural similarities that traditional sequence alignments might miss. This approach allows for a more nuanced understanding of protein functions across different subcellular localizations. By leveraging structural similarities that traditional sequence alignments might overlook, VAST enhances the robustness of predictions and facilitates a deeper understanding of protein functions across different subcellular localizations, offering a more nuanced insight into their biological roles and interactions.

Combinatorial extension (CE) algorithms are widely used in structural bioinformatics for the alignment of protein structures. The CE algorithm identifies the optimal alignment of two protein structures by comparing their spatial arrangements of alpha‐carbon atoms rather than their sequences. The process involves breaking down the protein structures into smaller segments and finding an optimal path that maximizes the alignment of these segments. The method emphasizes geometric similarity and is particularly useful for detecting evolutionary relationships between proteins with low sequence similarity but high structural resemblance. CE is computationally efficient and robust, making it an important tool in structural genomics and protein function prediction.

### 2.4 Profile‐Based Alignment Tools

HHblits stands out as a sensitive method for searching sequence databases, employing HMM–HMM comparison techniques that excel in detecting remote homologs with low sequence similarity. This method uses profile‐profile comparisons and iterative refinement strategies to enhance the accuracy and relevance of search results. Notable implementations of HHblits include SeqVec [62], which models protein sequences using deep learning, PSCL [63] for effective protein structure classification, and SWISS‐MODEL [64], a tool that integrates seamlessly with protein databases to facilitate model building and parameter optimization. These applications highlight the robustness and versatility of HHblits in bioinformatics research.

PSI‐BLAST is a powerful tool that performs sequence database searches by iteratively constructing a position‐specific scoring matrix (PSSM) from the results of initial searches. This iterative process allows for the refinement of searches with each round, enhancing the accuracy and specificity of the results. The method's efficacy is highlighted in various applications such as ML‐FGAT [16], which leverages PSI‐BLAST for fine‐grained annotations, and SCLpred‐EMS [31] as well as SCLpred‐MEM [65], which both utilize the tool for subcellular localization prediction. These implementations demonstrate PSI‐BLAST's capability to assess statistical significance accurately, using metrics such as E‐values and bit scores, to ensure reliable and precise alignments.

SAM employs a combination of dynamic programming and heuristic methods, allowing it to incorporate additional information such as secondary structure predictions and evolutionary data into its analyses. This multifaceted approach is evident in various implementations, including rHAT [66], which utilizes SAM for robust sequence alignment, GraphMap [67], known for its fast and accurate long‐read alignment, and S‐conLSH [68], which enhances profile‐profile alignments. SAM's capability extends to iterative refinement and modeling of three‐dimensional protein structures, demonstrating its integration with a range of bioinformatics tools. This adaptability makes SAM a powerful platform for comprehensive bioinformatics analysis, contributing significantly to advancements in the field.

### 2.5 Genome Alignment Tools

MUMmer is a versatile alignment tool used for both nucleotide and protein sequences, excelling in identifying maximal unique matches between genomes. It plays a crucial role in genome assembly, genome rearrangement analysis, and comparative genomics studies. Notable versions and applications of MUMmer include MUMmer4 [69], which introduces optimized algorithms for rapid string matching and substring searches, and earlier iterations such as the one described in [70]. Additionally, MUMCo [71] leverages the capabilities of MUMmer to detect conserved regions, structural variations, and elucidate evolutionary relationships, showcasing its utility in genomic research and its efficiency in handling large‐scale genomic data.

LAST employs an index‐based approach, efficiently searching for sequence similarities, which is crucial for applications such as genome assembly, sequence homology detection, and metagenomic analysis. This method is enhanced by the use of the seed‐and‐extend algorithm, facilitating the detection of both local and global similarities between sequences across multiple alignment modes. Key implementations of LAST include LAST‐TRAIN [72], which optimizes the parameters for similarity searches, as well as the integration of LAST with MAFFT for improved MSAs [73]. Additionally, LASTM [74] exemplifies the versatility and robustness of LAST in handling complex genomic data sets, showcasing its comprehensive utility in genomic research.

Bowtie is a memory‐efficient tool specifically designed for aligning short DNA sequences to large reference genomes. This tool utilizes an efficient seed‐and‐extend algorithm, which ensures rapid and accurate alignment, making it particularly suitable for high‐throughput sequencing applications. Notable implementations include the use of Bowtie and its more advanced version, Bowtie2, which are highlighted in studies such as [75, 76]. Additionally, FSVA [77] leverages Bowtie's capabilities in its workflows, demonstrating the tool's integration with downstream analysis tools. These features make Bowtie a cornerstone in genomic research, facilitating a wide range of genomic studies and applications.

## 3 Methodology

### 3.1 Dataset Creation

The data is taken from UniProt knowledge‐base 2019 [78] which is a freely accessible database of proteins. UniProt 2019 is equipped with recent experimentally derived protein sequences and their annotations. The dataset initially contained 190 192 protein sequences that were theoretically annotated for subcellular localization. However, some proteins in this dataset didn't have a specified location. Ultimately, only 155 143 proteins were accurately annotated with their subcellular localization. The numbers further decreased to 151 425 protein sequences because only those with a length of 30 amino acids or more were chosen. After applying homology reduction, the dataset was further reduced to 11 825 protein sequences. The initial set of proteins underwent redundancy reduction using an expectation (e‐value) threshold of 0.001. This means that no two sequences should be similar to each other with an expectation value of 0.001 or smaller. This redundancy reduction process also automatically removes any potential exact duplicates (identical sequences) from the dataset.

The final reduced dataset, consisting of 11 825 examples, is divided into eight separate classes i.e. Other, membrane, Cytoplasm, Golgi apparatus, Mitochondrion, Nucleus, Plastid, and Secreted. Each protein is assigned to one of the ten classes, and proteins associated with two or more classes are not included in our dataset. Also, for the purposes of better analysis, the ER is grouped with other membrane‐associated structures into a single “membrane” category. This decision was driven by the relatively low incidence of proteins exclusively localized to the ER in our datasets, which limited the statistical power and reliability of the predictions. By combining it with similar subcellular localizations, we aimed to enhance the robustness and interpretability of our results. The dataset is split into three sets using a ratio of 3:1:1. This means there are 7095 proteins in the training set, 2365 in the test set, and 2365 in the validation set. To ensure equal representation of all classes and avoid biases, the sets are split in an interleaved manner, ensuring each stage of model training includes a balanced distribution of classes i.e. The first three proteins are placed in the training set, the fourth protein is moved to the test set, the fifth one is moved to the validation set, and then the cycle repeats until all 11 825 proteins are fully divided. Table 1 presents details of class sizes.

**TABLE 1:** Data statistics of protein sequences across eight classes in training, test, and validation sets.

Class name	Training	Test	Validation	Total
Other	964	318	537	1819
Membrane	1543	537	292	2372
Cytoplasm	922	324	344	1590
Golgi apparatus	53	18	22	93
Mitochondrion	534	117	133	784
Nucleus	1307	430	441	2178
Plastid	467	140	136	743
Secreted	1451	481	460	2392

### 3.2 Without Alignments Protocol

For without alignment assessment, the sequences are encoded using one‐hot encoding. This means that each character in the sequences is converted into a numerical representation. One‐hot encoding is preferred for non‐numerical data because it is straightforward and doesn't depend on the specific similarities between characters. Also, characters can be difficult for machine‐learning models to handle. Each amino acid in a protein sequence is represented by a binary value ranging from 1 to 21 bits.

### 3.3 With Alignments Protocol

For the purpose of with alignments assessment, we created MSAs for all datasets by running PSI‐BLAST [79] iteratively with an e‐value of 0.001. In other words, we generated alignments of multiple homologous (but unannotated) sequences to capture evolutionary information. These alignments are encoded into MSA profiles by calculating frequencies of residues and gaps. The frequencies of the amino acids present in the original sequence were then clipped to 1 i.e. each amino acid is represented by a vector containing 22 frequencies corresponding to the amino acid types found in the list of homologous sequences [80].

Similarity has been assessed by BLAST [81] alignment.

### 3.4 Predictive Model

**FIGURE 1:**
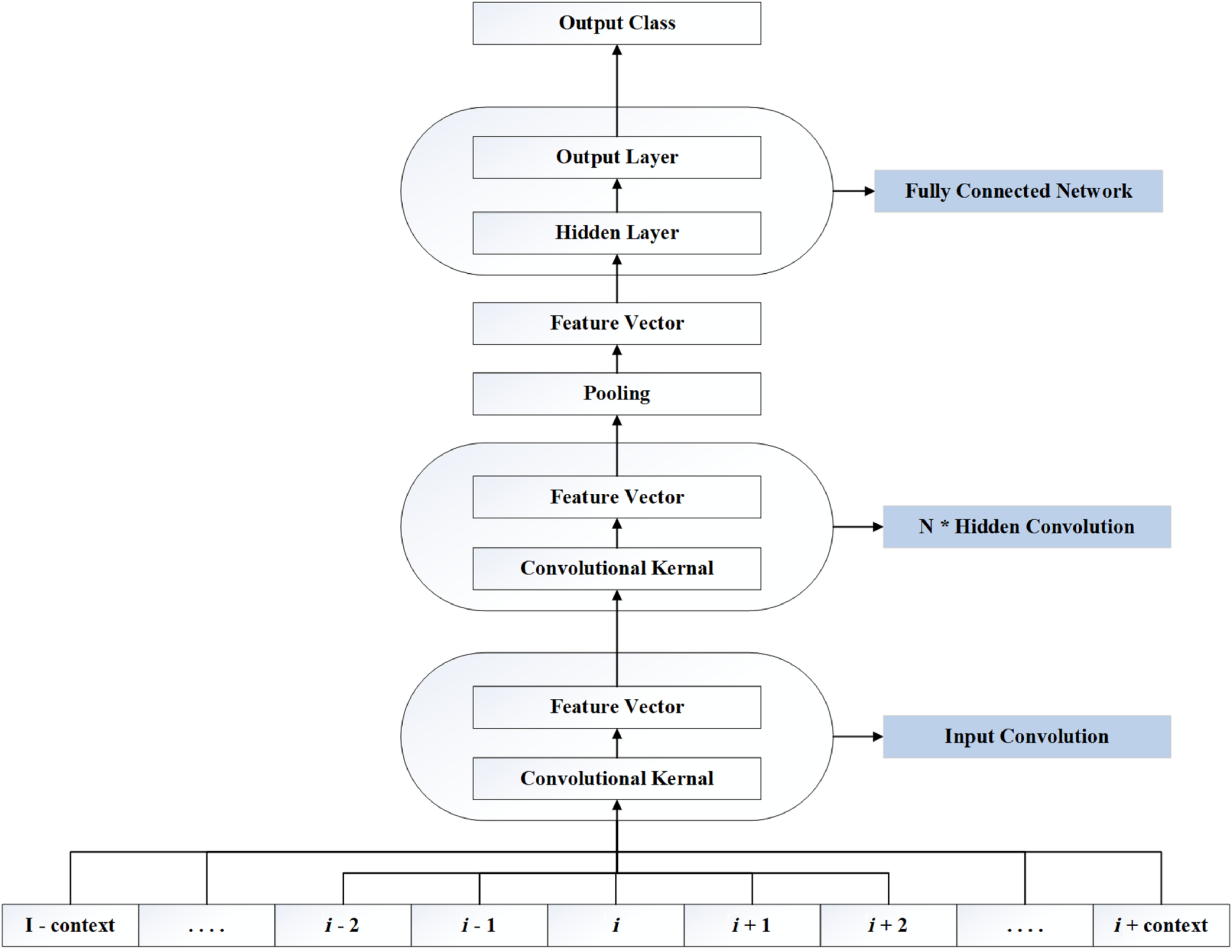
Predictive model: Deep N‐to‐1 convolutional architecture.

The predictive Model used is based on deep N‐to‐1 convolutional architecture. The N‐to‐1 Neural Network, or N1‐NN, is illustrated in Figure 1. The model is based on our framework to design neural networks specifically for structured data [82, 83]. The framework and architecture are explicitly explained in [84]. Deep N‐to‐1 CNNs are composed of an input kernel mapping a window of amino acids into a feature vector followed by a stack of hidden convolutional kernels followed by an average pooling unit over the whole sequence, and a final fully connected network [85]. A complete visualization of the predictive model is given in Figure 1.

As illustrated in Figure 1, predictive model layers are explained as follows:

*Input Kernel*: The first layer, referred to as the encoding layer, maps each amino acid into a hidden representation. When processing individual amino acids, it essentially performs compression and may uncover correlations or similarities between different types of amino acids in the process. When it processes a window of amino acids (the central amino acid along with C neighbors on either side), it embeds motifs into a compressed space instead of individual amino acids. The layer considers motifs of length 2×C+1, whether it operates on single amino acids or motifs. Its outputs can be assembled by downstream kernels to consider motifs or expand the size of those considered by the first layer. All replicas of this input kernel (equal to the length of the protein) may be processed in parallel. In our model, this initial layer is a 2‐layered neural network to leverage the universal approximation properties of neural networks. This choice increases the minimal depth of the model, potentially making it more powerful but also harder to train effectively.

*N* × *Hidden Convolution*: After the initial kernel, feature extraction stages follow. Each feature extraction stage $N_{i}$ uses a convolution from i−c to i+c hidden states to derive a new vector from the previous hidden states $N_{i-1}$. This process continues, with each stage deriving from the previous one, effectively increasing the context size. The effective context size is $\mathrm{cs}=\left(2c\times N\right)+1$, where c is half the convolution window size and N is the network depth. As the number of stages increases, the motifs considered become larger, so reducing the context size per stage might be necessary to target an optimal motif size.

*Pooling*: After reaching the last feature vector, it is pooled to form the final output layer, which serves as input to the fully connected network via a many‐to‐1 relationship. This feature vector v compresses sorting motifs into j abstract descriptors, learned to minimize classification error. These descriptors are property‐driven, depending on both the input sequence and the classification target used during training.

*Fully Connected Network*: A fully connected neural network processes the global feature vector v, mapping it to the corresponding output label. The output is then transformed by a softmax function, normalizing it into class probability estimates. The label with the highest probability is chosen as the model's prediction, reflecting the model's confidence.

The term “predictive model” in our study refers to the Deep N‐to‐1 CNN architecture we specifically designed to evaluate the impact of sequence alignments on prediction accuracy for protein subcellular localization. This model was tailored to provide a clear comparison between alignment‐based and alignment‐free approaches under consistent experimental conditions, ensuring that differences in performance could be attributed directly to the presence or absence of alignments rather than to variations in model design.

To position our predictive model against existing CNN architectures and other machine‐learning approaches, we conducted extensive experimental benchmarking. Our approach is carefully calibrated, with hyperparameters optimized through grid search techniques, ensuring that the model is functioning at its best possible performance level. While we acknowledge that there are various CNN models and other machine learning techniques, including SVMs, Random Forests, and Transformer‐based models, our N‐to‐1 CNN architecture was chosen due to its unique capability to handle diverse data without requiring the pre‐processing complexities of alignment generation.

Regarding the model's comparative performance, while direct benchmarking against every alignment‐free method in the literature is impractical due to differences in datasets and experimental setups, our results (Section 4) showed consistent and significant improvements in prediction accuracy compared with traditional methods reported in similar contexts. However, we recognize the challenge of definitively stating superiority over all alignment‐free methods without a direct, large‐scale comparison study. Our model's demonstrated robustness across varied subcellular localization classes suggests its high potential, but further comparative studies using standardized datasets would provide more comprehensive validation of its relative performance.

### 3.5 Training and Ensembling

The models undergo training and tuning using a combination of train, test, and validation datasets. During training, stochastic gradient descent is employed to minimize the relative entropy between the model's output and the target. The models are trained over 10 epochs, with each epoch covering the entire training set. After training, they are tested on a validation set. The validation set is employed to direct the training process, helping to select models and evaluate for potential over‐fitting. Throughout this process, the decreasing error and other relevant results are recorded in a log file. Additionally, the model parameters are saved separately for future use. This process repeats 10 times per epoch, for a total of 5000 epochs. This strategy results in the creation of 500 models that are interconnected yet distinct from each other. From the pool of 500 models, the six models exhibiting the highest performance on the validation set are selected. Performance evaluation is briefly covered in Section 4.

### 3.6 Hyperparameters Tuning

Different trainings are conducted in a preliminary stage, searching for good sets of hyperparameters. For hyper‐parameters, a grid search approach is adopted for fine‐tuning, applied to all datasets under consideration. Hyper‐parameters used are given in Table 2. A thorough hyperparameter search is essential for enhancing the model's ability to generalize effectively. To find the best possible hyperparameters, initially, a comprehensive hyperparameter search was conducted using the first fold of the dataset to identify the optimal model (model selection). Also, we tested configurations of Deep N‐to‐1 CNNs of various depths and widths during preliminary experiments to get a sense of better‐performing values. In short, multiple training sessions were conducted with varying hyperparameters to identify better‐performing parameter values. This process helped to narrow down an optimal range of hyperparameters for further experimentation. The hyperparameters were then adjusted based on the performance of the validation dataset. Fully tuned hyperparameters are mentioned in Table 2. In Table 2, hyperparametric values of ε, NHt, Ht, NHi are kept uniform throughout the experimentation i.e. ε (0.02), nEpochs (5000), NHt (20), Ht (15), NHi (15). However, NL and γ are changed to draw various interpretations mentioned in Section 4.

**TABLE 2:** List of hyperparameters for both with alignments and without alignments experimentation.

Hyperparameter name	Abbreviation	Description
Learning rate	ε	It is the learning rate in our experiments typically set at 0.02.
Number of layers	NL	This is the number of hidden‐to‐hidden convolutional stages. A variable number of hidden‐to‐hidden layers i.e. 6, 8, 10 are used.
Kernel sizes	γ	It is semi‐context of inputs. Experiments are using various γ sizes ranging from 0 to 20.
Number of epochs	nEpochs	The process repeats epochs/10 times for a total of 5000 epochs.
Number of hidden units	NHt	It is the number of the hidden units in the hidden‐to‐hidden convolutional stages and set at 20 for each layer.
Number of units in the output	Ht	It is the number of units in the output of the hidden‐to‐hidden convolutional stages and set at 15 for each layer.
Number of hidden units in the input	NHi	It is the number of hidden units in the input‐to‐hidden convolutional stage and set at 15 for each layer.

### 3.7 Performance Evaluation

To evaluate the performance of a specific class, we calculated Matthew's correlation coefficient (MCC), accuracy (ACC), specificity (Spec), sensitivity (Sens), and F1‐score based on the following formulas.

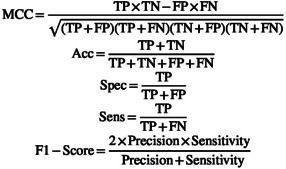

where true positives (TP): the number of sequences predicted to be in a specific class that is actually observed in that class. False positives (FP): the number of sequences predicted to be in a specific class that is not actually observed in that class. True negatives (TN): the number of sequences predicted not to be in a specific class that are actually not observed in that class. False negatives (FN): the number of sequences predicted not to be in a specific class that is actually observed in that class.

In order to evaluate the performance (P) of models, the equation below is used:

$$P=\frac{\sum_{i}Z_{\mathrm{ii}}}{\sum_{\mathrm{ij}}Z_{\mathrm{ij}}}$$

where P is the performance of models. True positives (TP): $z_{\mathrm{ii}}$. False positives (FP): $\sum j\neq i\;Z_{\mathrm{ji}}$. True negatives (TN): $\sum v\neq i\sum j\neq i\;Z_{\mathrm{jv}}$. False negatives (FN): $\sum j\neq i\;Z_{\mathrm{ij}}$.

Each hidden convolutional kernel learns a non‐linear function $H^{k}$ at hidden layer k from a window of intermediate states at position j and predicts an intermediate state vector *i* at position i.

## 4 Results And Analysis

We experimented with different kernel sizes, defined as the context layer or semi‐context of inputs, denoted by gamma (γ), for hidden layers. This involves taking inputs by considering the size of context layers for hidden‐to‐hidden networks. Accordingly, the number of inputs by the kernels is 2×γ+1. For our results and analysis, various γ sizes were trained, tested, and validated to evaluate their effectiveness with larger datasets and a greater number of classes for alignments versus without alignments interpretations. We tested γ values ranging from 0 to 20, observing varied training patterns across different sizes of layers with alignments versus nonalignments cases.

It is also interesting to note that, unlike standard CNNs, all convolutional kernels and the final fully connected networks are implemented using feed‐forward neural networks (FFNN) with two hidden layers. Each convolutional kernel consists of two layers: an initial kernel layer followed by a nonlinearity, then another kernel of size 1 followed by another nonlinearity. We use sigmoidal nonlinearities for the model's internal units instead of rectified linear units. Preliminary tests indicated that deeper convolutional stages and sigmoidal units were beneficial. Consequently, the architecture has a minimum of three internal hidden layers when no hidden‐to‐hidden convolutional kernel is present. An architecture with k hidden‐to‐hidden kernels has a total of 3+2k hidden layers.

**FIGURE 2:**
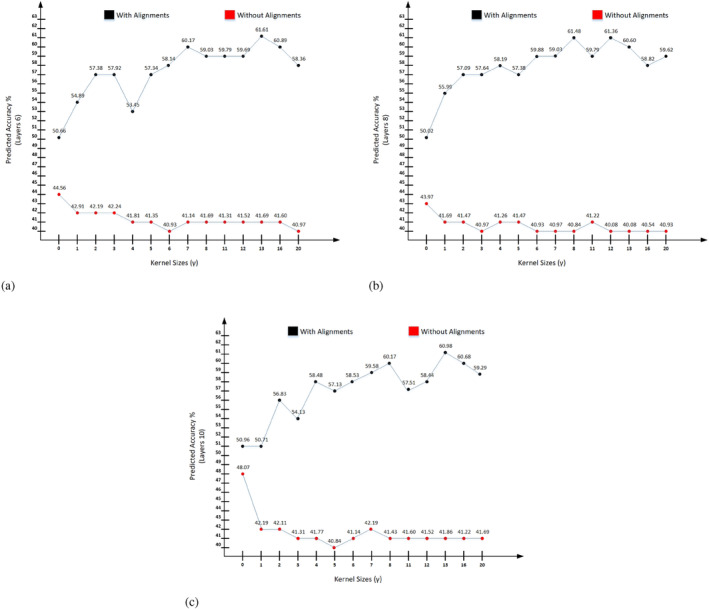
Prediction accuracies with respect to various network layers and kernel sizes, both with alignments and without alignments.

- The Figure 2a illustrates the predicted accuracies for various kernel sizes, comparing models with alignments and without alignments for six network layers. For kernel size 1, the accuracy rises to 54.89%, a significant increase from the baseline. This trend continues with kernel size 2, where the accuracy further improves to 57.38%. The accuracy peaks at 57.92% for kernel size 3, showing an upward trend in the early stages and so on. In contrast, the model without alignments exhibits a decline in performance, with accuracies dropping to 42.91% and 42.19% for kernel sizes 1 and 2, respectively. By kernel size 3, the accuracy slightly increases to 42.24%, but still remains significantly lower than the aligned model throughout the next kernels.
- The Figure 2b illustrates the predicted accuracies for various kernel sizes, comparing models with alignments and without alignments for eight network layers. As kernel sizes increase, the model with alignments shows a consistent improvement in accuracy. At kernel size 1, the accuracy jumps to 55.99%, a substantial increase from the baseline. This upward trend continues, reaching 57.09% at kernel size 2 and 57.64% at kernel size 3 and so on. In contrast, the model without alignments demonstrates a decline, dropping to 41.69% at kernel size 1 and further to 41.47% at kernel size 2. By kernel size 3, the accuracy without alignments falls to 41.26%, highlighting a growing disparity between the two models.
- The Figure 2c illustrates the predicted accuracies for various kernel sizes, comparing models with alignments and without alignments for ten network layers. As kernel sizes increase, the model with alignments demonstrates consistent improvement. For kernel size 1, the accuracy decreases slightly to 50.71%, but then significantly increases to 56.83% for kernel size 2. Further improvements are seen with kernel sizes 3 and 4, where accuracies reach 54.13% and 58.48%, respectively. In contrast, the model without alignments shows a consistent decline in performance, with accuracies of 42.19%, 42.11%, and 41.31% for kernel sizes 1, 2, and 3, respectively, stabilizing at lower values.

As illustrated in Figure 2a, the graph effectively illustrates the significant difference in model performance with and without alignments. Alignments greatly enhance the model's accuracy, especially when combined with an optimal kernel size. In baseline comparison, with Alignments shows 50.66% and without alignments shows 44.56% prediction accuracy. Therefore, the baseline accuracy is higher with alignments by about 6.1%. However, analyzing the peak performances shows that with Alignments is 61.61% (Kernel Size 11) and without alignments is 44.56% (Kernel Size 0). The highest accuracy achieved with alignments is significantly higher by 17.05% compared with the highest without alignments. Overall, with alignments graph trend shows significant variability across kernel sizes, with several peaks and troughs. The overall trend shows substantial improvement in accuracy as kernel size increases, peaking at kernel size 11. However, without alignments graph line shows the accuracy remains relatively stable and low, fluctuating slightly but showing no substantial improvement with increasing kernel sizes. The differences between the highest and lowest accuracies are minor and steady depicting the lag phase.

As illustrated in Figure 2b, the baseline comparison shows that the model with alignments starts with higher accuracy and continues to improve significantly with increasing kernel sizes. The peak performance of the aligned model at 61.48% for kernel size 11. However, without alignments trend shows contrasts with the stable but lower performance of the model, which peaks at only 43.97% at kernel size 0 and continues to show a lag phase with a difference of only a point i.e. rest of all the prediction accuracies lies between 40.08% and 41.69% which shows a very steady graph line. For example, for kernel sizes beyond 12, the aligned model's accuracies slightly decrease but remain high, with 60.60% at kernel size 16 and 59.62% at kernel size 20. The model without alignments, however, continues to display minimal variation, with accuracies hovering around 40.08%–40.97%, reinforcing its consistently lower performance. The upward trend of with alignment ratios highlights the model's responsiveness to changes in kernel size. In contrast, the model without alignments exhibits a decline in performance, with accuracies of 41.69%, 41.47%, and 41.26% for the same kernel sizes, respectively. This indicates a growing disparity as the kernel sizes increase.

As illustrated in Figure 2c, the model with alignments starts with higher accuracy and continues to improve significantly with increasing kernel sizes. The peak performance of the aligned model at 60.98% for kernel size 12. However, the nonalignment trend line shows contrasts with the stable but lower performance of the model, which peaks at only 48.07% at kernel size 0 and continues to decline. Apart from the declining graph, the accuracy remains under 2 points of a difference i.e. 40.84%–42.19%. The model with alignments demonstrates consistent improvement. For kernel size 1, the accuracy decreases slightly to 50.71%, but then significantly increases to 56.83% for kernel size 2. Further improvements are seen with kernel sizes 3 and 4, where accuracies reach 54.13% and 58.48%, respectively. In contrast, the model without alignments shows a consistent decline in performance, with accuracies of 42.19%, 42.11%, and 41.31% for kernel sizes 1, 2, and 3, respectively, stabilizing at lower values (minimal variation). The most significant improvement with alignment is observed at kernel sizes 12 and 13, where the accuracies reach their peak at 60.98% and 60.68%, respectively. However, without alignments graph line shows a slight improvement at kernel size 7 with an accuracy of 42.19%, but this is still considerably lower than the peak performance of the aligned model.

We can access from Figure 2b that the highest accuracy achieved with alignments is 61.48% (at kernel size 12), while the highest accuracy without alignments is 43.97% (at kernel size 0). Therefore, the highest accuracy achieved with alignments is significantly higher by 17.51% compared with the highest without alignments. Also, Figure 2c demonstrates that the highest accuracy achieved with alignments is 60.98% (at kernel size 12), while the highest accuracy without alignments is 48.07% (at kernel size 0). Therefore, the highest accuracy achieved with alignments is significantly higher by 12.91% compared with the highest without alignments. Also, Figure 2a illustrates that the highest accuracy achieved with alignments is 61.61% (Kernel Size 11) and without alignments is 44.56% (Kernel Size 0). The highest accuracy achieved with alignments is significantly higher by 17.05% compared with the highest without alignments. We observe that the models incorporating alignments consistently achieve significantly higher prediction accuracies compared with those without alignments. Specifically, the highest accuracies achieved with alignments are 61.61%, 61.48%, and 60.98% respectively, while the highest accuracies without alignments are considerably lower at 44.56%, 43.97%, and 48.07%. The average difference in peak performance for all layers was calculated at approximately 15.82%.

Table 3 shows detailed stats for eight classes with alignments (Table 3a) and without alignments (Table 3b). Table 3 tells about performance per class based on (MCC) is Matthew's correlation coefficient, accuracy, specificity, and sensitivity. Also, the F1‐score measures the predictive performance per class. Table 3a, shows significantly higher performance across all metrics and classes. Particularly, classes such as “Membrane,” “Nucleus,” and “Secreted” benefit the most from alignments. Table 3, noticeable drop in performance metrics across all classes. The “Golgi Apparatus” class is not predicted at all without alignments. With alignments stats given in Table 3a indicate, the “Other” class shows low overall performance, with an accuracy of 26.74% and an F1 score of 17.89%. In contrast, the “Membrane” class exhibits high performance, achieving an accuracy of 71.67% and an F1 score of 77.89%. This indicates the model's effectiveness in predicting the “Membrane” class with alignments. The “Cytoplasm” class has moderate performance, with an accuracy of 44.21% and an F1 score of 47.59%, showing reasonable specificity but lower sensitivity. The “Golgi Apparatus” class, however, displays poor performance with an accuracy of only 1.35% and an F1 score of 27.00%, despite high specificity. The “Mitochondrion” class performs better, with an accuracy of 53.33% and an F1 score of 50.45%. The “Nucleus” class demonstrates strong performance with an accuracy of 55.68% and an F1 score of 65.19%. Similarly, the “Plastid” class shows good performance, achieving an accuracy of 57.50% and an F1 score of 52.47%.

**TABLE 3:** The table presents performance statistics for protein subcellular localization prediction across eight classes, comparing classes with and without alignments.

Subcellular class	MCC	Accuracy	Specificity	Sensitivity	F1 Score
(a) Detailed stats for eight classes with alignments.					
Other	0.10	26.74%	86.99%	26.74%	17.89%
Membrane	0.72	71.67%	96.02%	71.67%	77.89%
Cytoplasm	0.40	44.21%	93.34%	44.21%	47.59%
Golgi apparatus	0.19	1.35%	92.31%	26.67%	27.00%
Mitochondrion	0.48	53.33%	97.43%	53.33%	50.45%
Nucleus	0.58	55.68%	95.66%	55.68%	65.19%
Plastid	0.50	57.50%	97.12%	57.50%	52.47%
Secreted	0.80	78.98%	97.53%	78.98%	84.05%
(b) Detailed stats for Eight classes with no alignments.					
Other	0.04	19.44%	86.73%	19.44%	11.22%
Membrane	0.36	69.55%	78.14%	69.55%	45.31%
Cytoplasm	0.16	27.40%	89.56%	27.40%	26.06%
Golgi apparatus	0.06	0.00%	99.27%	0.00%	0.00%
Mitochondrion	0.03	2.14%	95.66%	2.14%	3.09%
Nucleus	0.42	43.52%	92.85%	43.52%	52.93%
Plastid	0.17	24.39%	95.05%	24.39%	21.74%
Secreted	0.53	51.98%	94.46%	51.98%	62.55%

*Note*: The metrics considered are MCC, accuracy, specificity, sensitivity, and F1 score.

The “Secreted” class stands out with excellent performance, achieving an accuracy of 78.98% and an F1 score of 84.05%, indicating a highly effective model for this class with alignments. There are several possible reasons about why our predictive model performed particularly well for this class. First, secreted proteins often possess distinct sequence signals, such as signal peptides, that are easier to identify compared with other localization signals. These signal peptides are well‐characterized, making it simpler for predictive models to detect and classify them accurately. The presence of these unique and highly conserved sequence motifs provides strong cues that improve model accuracy. Second, our training dataset include a substantial representation of secreted proteins, which may contribute to the model's higher performance for this category. The availability of high‐quality, annotated data for secreted proteins allows the model to learn more effectively, resulting in improved predictive accuracy. Finally, secreted proteins typically follow more predictable pathways compared with other subcellular locations where localization signals can be more ambiguous or complex. This predictability enables the model to achieve higher accuracy as it can better generalize learned features from training data.

However, Table 3b, indicates the opposite situation. The model without alignments exhibits generally lower performance across all metrics and classes. The “Other” class shows even poorer performance, with an accuracy of 19.44% and an F1 score of 11.22%. The “Membrane” class, while maintaining relatively high accuracy at 69.55%, experiences a drop in performance without alignments, with a lower F1 score of 45.31%. The “Cytoplasm” class sees a significant drop in performance, achieving an accuracy of 27.40% and an F1 score of 26.06%. Notably, the “Golgi Apparatus” class is not predicted at all without alignments, resulting in zero accuracy and F1 score. The “Mitochondrion” class shows extremely poor performance without alignments, with an accuracy of 2.14% and an F1 score of 3.09%. The “Nucleus” class maintains moderate performance, but with noticeable declines, achieving an accuracy of 43.52% and an F1 score of 52.93%. The “Plastid” class also shows lower performance metrics without alignments, with an accuracy of 24.39% and a F1 score of 21.74%. The “Secreted” class still shows good performance, but all metrics are lower compared with alignments, with an accuracy of 51.98% and an F1 score of 62.55%. Based on the above stats, the average difference in the highest accuracy achieved with alignments compared with without alignments is approximately 15.16%.

Overall, graphs illustrate the impact of alignments on the predictive accuracy of protein subcellular localization models across different layers and kernel sizes. From a critical standpoint, the noticeable gap in accuracy between models with and without alignments emphasizes the role of evolutionary information captured through alignments in enhancing model performance. This suggests that sequence alignments contribute significantly to the model's ability to capture sequence conservation patterns that are pivotal for accurate subcellular localization. Despite varying kernel sizes and depths, models using alignments consistently outperform those without, highlighting a clear dependency on alignment‐derived features that may not be easily replicated by alternative encoding methods.

Furthermore, the performance of models without alignments remains relatively flat across varying kernel sizes, indicating limited sensitivity to architectural changes when alignment information is absent. This stability could reflect an inherent limitation in the ability of nonalignment‐based models to learn complex patterns purely from raw sequence data, which may lack the contextual and structural depth provided by alignments. Such findings point toward a fundamental gap in predictive capability when critical sequence relationships are not leveraged, underscoring the importance of exploring supplementary methods or hybrid approaches that can bridge this accuracy gap without the computational overhead of full alignments. The results provoke deeper questions about the balance between computational efficiency and biological accuracy in protein subcellular localization tasks.

## 5 Conclusion

Subcellular localization prediction is crucial for understanding protein function and cellular processes. By investigating the impact of alignments on prediction accuracy, we can conclude that the higher accuracy achieved with alignments indicates that our predictive model can make more precise predictions, which is essential for applications where predictive accuracy is critical. Also, the consistent improvement across various kernel sizes and layers shows that the alignment technique enhances the model's robustness, making it less sensitive to changes in configuration parameters. Higher accuracy with alignments implies a more reliable model, which can be trusted to deliver consistent performance across different layers and dimensions of accuracy predictions. The significant difference among classes considered highlights the importance of alignments in enhancing the predictive performance of the model across various subcellular classes as well. This enhancement is crucial for developing accurate, robust, and reliable predictive models, making it a significant consideration in the field of protein subcellular localization predictions.

We conclude that alignment‐free approaches may struggle to capture the full spectrum of information that traditional alignment‐based methods offer due to several inherent limitations. Alignment‐free methods do not integrate structural information, which limits their ability to understand sequence‐structure relationships. In contrast, alignment‐based techniques often utilize sophisticated scoring matrices and statistical methods to assess sequence matches, allowing for the identification of significant evolutionary and structural relationships. This provides a level of statistical rigor that helps to reduce false positives or negatives in predictions. The global perspective offered by alignment‐based methods enables a comprehensive comparison of entire sequences, revealing long‐range dependencies and interactions that are often missed in alignment‐free methods. Consequently, while alignment‐free approaches are advantageous in terms of computational speed and applicability to large‐scale datasets, they fall short in replicating the detailed, functionally relevant insights that alignment‐based methods provide, impacting the overall accuracy and reliability of protein subcellular localization predictions. Also, evolutionary context is largely absent in alignment‐free methods, which typically rely on sequence composition, word frequencies, or k‐mer distributions. As a result, these approaches may miss conserved motifs that are indicative of functional or structural similarities, especially in cases of remote homology where sequence identity is low but functional similarity remains significant. The absence of gap and index analysis in alignment‐free methods further restricts their capacity to capture important evolutionary changes, which can play a crucial role in subcellular localization and function prediction.

## Author Contributions

**Maryam Gillani:** conceptualization, investigation, funding acquisition, writing – original draft, writing – review and editing, validation, methodology, visualization, software, formal analysis, resources, data curation. **Gianluca Pollastri:** conceptualization, investigation, writing – review and editing, visualization, validation, methodology, software, formal analysis, project administration, resources, supervision, data curation, writing – original draft.

## Consent

The authors have nothing to report.

## Conflicts of Interest

The authors declare no conflicts of interest.

## Data Availability

The data that support the findings of this study are openly available in Index of /SCL8 at http://distilldeep.ucd.ie/SCL8/.
